# Low-Cost Magnesium-Based Thermoelectric Materials: Progress, Challenges, and Enhancements

**DOI:** 10.1021/acsaem.4c00961

**Published:** 2024-07-09

**Authors:** Zhenxue Zhang, Mikdat Gurtaran, Hanshan Dong

**Affiliations:** School of Metallurgy and Materials, University of Birmingham, Birmingham B15 2TT, United Kingdom

**Keywords:** magnesium, thermoelectric, degradation, coatings, contact

## Abstract

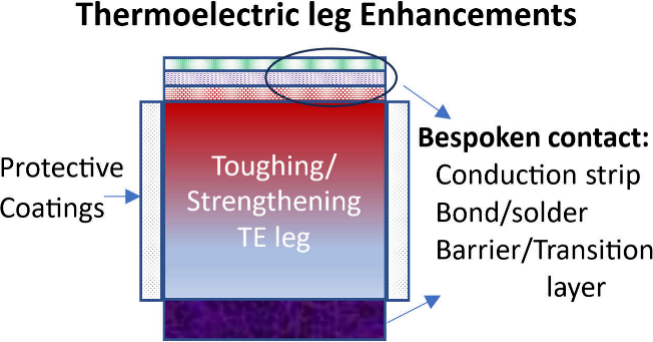

Magnesium-based thermoelectric (TE) materials have attracted considerable interest due to their high ZT values, coupled with their low cost, widespread availability, nontoxicity, and low density. In this review, we provide a succinct overview of the advances and strategies pertaining to the development of Mg-based materials aimed at enhancing their performance. Following this, we delve into the major challenges posed by the severe working conditions, such as high temperature and thermal cycling, which adversely impact the behavior and long-term stability of the TE modules. Challenges include issues like the lack of mechanical strength, chemical instability, and unreliable contact. Subsequently, we focus on the key methodologies aimed at addressing these challenges to facilitate the broader application of the TE modules. These include boosting the mechanical strength, especially the toughness, through grain refining and additions of second phases. Furthermore, strategies targeted at enhancing the chemical stability through coatings and modifying the microstructure, as well as improving the contact design and materials, are discussed. In the end, we highlight the perspectives for boosting the practical applications of Mg-based TE materials in the future.

## 1 Introduction

The depletion of natural resources and environmental degradation necessitate the adoption of clean and renewable energies to reduce dependence on fossil fuels. Simultaneously, a significant portion of the world’s energy is squandered in the form of heat emissions, including vehicle exhaust, industry steam boiler exhaust, overheated solar panels, and home appliances like air-conditioning/refrigeration condensers, ovens, and computers.^1^

Thermoelectric (TE) technology converts heat directly into electricity via the Seebeck effect, created by the temperature difference between hot and cold ends. This process requires nonmoving parts, emits zero-emission, and ensures long-steady operation, making it promising for waste heat retrieval, power generation, and refrigeration.^2^ Moreover, TE technology can enhance the efficiency of utilizing traditional fossil fuels by converting waste heat back into electricity, contributing to energy savings and environmental preservation. The merit of a TE material can be assessed by the dimensionless figure of merit, ZT = *S*^2^σ*T*/κ, where *S* stands for the Seebeck coefficient, σ is the electrical conductivity, *T* represents the temperature, and κ is the thermal conductivity.^2^

Theoretical calculations suggest the maximum ZT value can reach 14 for Bi_2_Te_3_-based nanowires materials.^3^ Additionally, thin-film Heusler alloys based on Fe_2_V_0.8_W_0.2_Al, fabricated by magnetron sputtering, can theoretically achieve a maximum ZT of 7 between 300 and 400 K.^4^ In practical form, the ZT values of the materials have been significantly improved toward 3 via techniques such as band engineering, doping, and artificial micro–nanostructure processing by modulating the thermal conductivities, electrical conductivities, and Seebeck coefficients of the materials. Experimental measurements have shown that the ZT value of N-type SnSe hit 2.8 at 773 K.^5^ Layered flake Cu_1.94_Al_0.02_Se, prepared by DC hot pressing process, obtained a ZT value of 2.62 at 1029 K.^6^ Kim et al. claimed a high ZT of 1.86 at 320 K for Bi_0.5_Sb_1.5_Te_3_ synthesized by liquid-phase compression.^7^ Significant advances have been made in TE materials with high performance across a wide temperature range since the early 2000s.^8,9^

Traditional TE materials with high ZT values include Bi_2_Te_3_, Sb_2_Te_3_ materials suitable for low-temperature range (<523 K), PbTe and TAGS-85 materials utilized in midtemperature range (523–823 K), and SiGe materials employed in high-temperature range (>823 K).^1,10^ Other high performance TE materials include skutterudites materials, Mg_2_Si, high manganese silicides, half-Heusler alloys, Zintl compounds, sulfides, and selenides, as well as multicomponent oxides series and organic–inorganic composites series.^11−14^ However, these compounds often contain toxic elements like Pb and expensive, rare elements such as Te, In, Hf, and Bi. Additionally, common PbTe, CoSb_3_, and Bi_2_Te_3_ have high mass densities between 6.5 and 8.5 g·cm^–3^, potentially hindering their application, particularly in industries like automotive and aerospace.^15^ Attributes such as low density, plastic deformation, and high fracture toughness^16^ are critical for TE applications to withstand mechanical stress from vibrations and thermal cycling.^17^

Magnesium-based TE materials offer a promising alternative, utilizing less expensive, nontoxic, and earth-abundant materials to meet environmental regulations and gain widespread acceptance in the energy market.^18^ While the current cost of TE system (above 10 $/W) is higher than other clean power-generation technologies like photovoltaics ($0.5/W) and wind power ($0.4/W),^19,20^ the cost of raw materials significantly impacts TE modules costs. Mg_2_Si_0.6_Sn_0.4_, for instance, has a material cost of about $4/kg, much cheaper than commercialized TE materials such as Bi_2_Te_3_, PbTe, and SiGe, with expenses of $110/kg, $81/kg, and $371/kg, respectively.^17^ Moreover, Mg-based materials have low density (ρ = 1.98–2.76 g·cm^–3^), providing an advantage in the specific figure of merit (ZT/ρ) over other commercial TE materials like Skutterudites and PbTe, crucial for applications where weight is a consideration, such as airborne and motion devices.^21^

Polymeris et al. reviewed the micro- and nanostructure properties, as well as the role of alloying in the development of Mg_2_Si based TE materials.^22^ Zhou et al. summarized the recent advances of Mg-based thermoelectric, including Mg_2_X (X = Si, Ge, Sn), Mg_3_(Sb,Bi)_2_, and α-MgAgSb, from both material and device level.^23^ They also analyzed the strategies to maximize their ZT values and the conversion efficiency from modifying their electronic band structures, crystal structures, and thermal and electrical transport properties. Similar principles for Mg_3_Sb_2_ and its derivatives was outlined by Shi et al.^24^ Han et al. summarized the progress of magnesium-based energy materials in 2023.^25^ However, there has been less emphasis on studying the mechanical and chemical stability of these materials.^22^ In this review, we first highlight recent advances in Mg-based TE materials (Section 2). Subsequently, we identify the major challenges and hurdles encountered in the application of TE techniques (Section 3), such as brittleness (Section 3.1), susceptibility to oxidation (Section 3.2), and high contact resistance (Section 3.3). We then outline proposed solutions to address these issues, which include toughing the TE materials through grain refinement and addition of second phases (Section 4.1), protecting the TE legs with various coatings (Section 4.2), improving contact by modifying the design and carefully choosing contact materials (Section 4.3), etc. After briefly introducing the progress of the magnesium-based thermoelectric generator (TEG) modules (Section 5), we finally conclude with some perspectives on the future development of Mg-based TE materials and modules (Section 6).

## 2 Development of the Low-Cost Mg-Based Thermoelectric Materials

**Figure 1:**
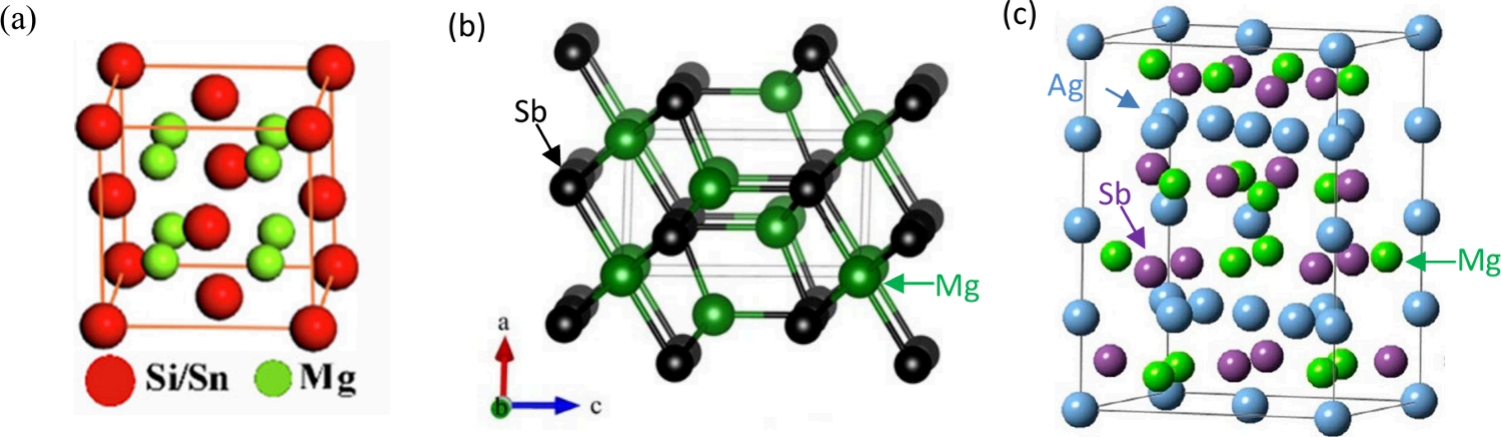
Crystal structure model of (a) Mg_2_Si or Mg_2_Sn,^30^ Reproduced from ref (30), Copyright [2020] [American Chemical Society]; (b) Mg_3_Sb_2_ (P3̅m1 phase),^31^ Reproduced from ref (31), Copyright [2020] [American Chemical Society]; and (c) α-MgAgSb.^32^ Reproduced from ref (32), Copyright [2015] [American Chemical Society].

The binary compounds of Mg_2_X (X: Si, Ge, Sn) exhibit a face-centered cubic (FCC) crystal structure, with the unit cell composed of 12 atoms.^26^ In this structure, the X^4–^ ions occupy the four face-centered cubic positions, while the Mg^2+^ ions occupy the eight centered tetrahedral sites (Figure 1a). Mg_2_Si possesses a narrow bandgap and exhibits N-type conductivity, primarily due to native defects. It demonstrated both a high electronic conductivity and a high Seebeck coefficient, with a peak figure of merit, ZT, ranging from 0.6 to 0.8.^27^ Additionally, its high melting point (∼1358 K) and excellent thermophysical properties, including boosted compression strength (1640 MPa) and Young’s modulus (120 GPa), along with a low thermal expansion coefficient, make it fit for a wide range of environmental applications.^22^ Alternatively, Mg_2_Sn and Mg_2_Ge have lower peak ZT value, which may not be sufficient for practical TE module applications.^28^ For instance, the maximum ZT for N-type Mg_2_Ge, fabricated through reaction of MgH_2_ and Ge via spark plasma sintering (SPS), reaches only 0.32.^29^

The intermetallic compounds such as Mg_2_Si, Mg_2_Ge, and Mg_2_Sn demonstrate higher thermal conductivity than those traditional BiTe-based and PbTe-based TE materials, which impose limitations on their maximum ZT value. The coupling between thermal and electrical transport properties makes it challenging to enhance the TE performance of Mg_2_X binary compounds, as these properties are directly influenced by carrier concentration. Furthermore, the concentration of majority carriers has essential influence on the electrical transport properties, especially at high temperatures where the bipolar effect may come to play. Mg_2_Si-based materials offer carrier controllability through impurity doping, providing design flexibility in adjusting thermal impedance. Thus, optimizing carrier concentration for maximal TE performance is vital, achievable through band structure engineering techniques such as doping the matrix with acceptor or donor elements to achieve band convergence, forming solid solutions, introducing point defects, and modifying the nanostructure to influence electrical conductivity and Seebeck coefficient.^23,33^

**Figure 2:**
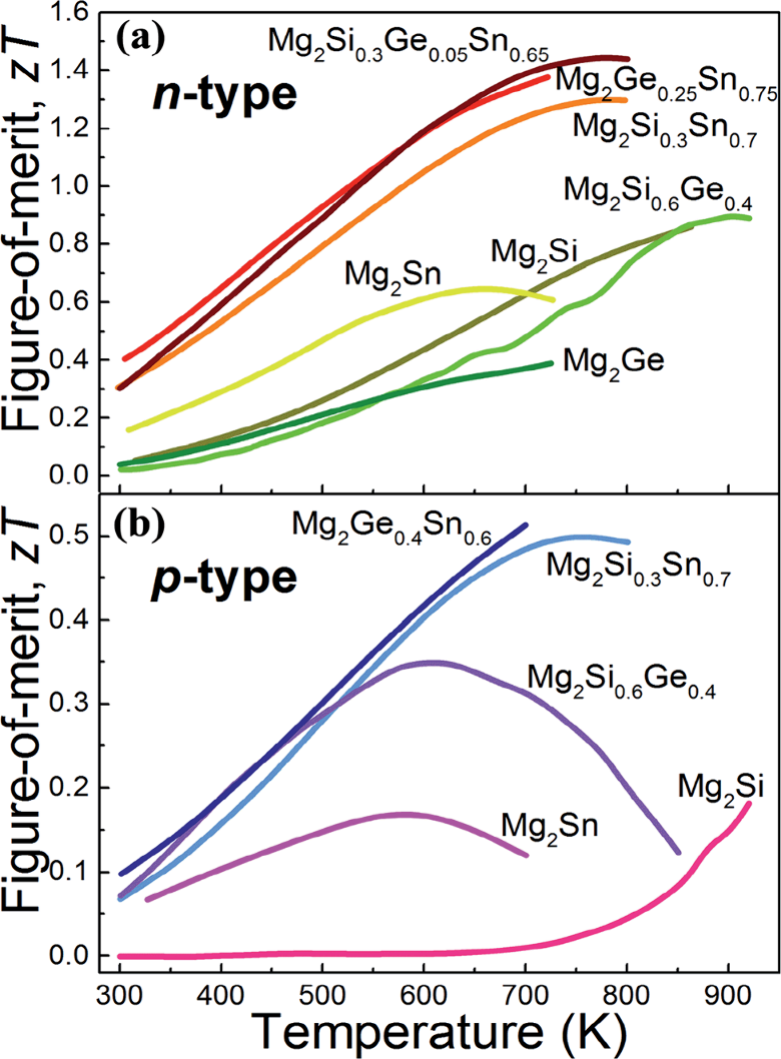
A summary of the development of (a) N-type and (b) P-type Mg-based TE materials with different alloying elements up to 2018.^17^ Reproduced with permission from ref (17), Copyright [2018], [RSC publishing].

Extensive interest and attention have been directed toward the development of the Mg_2_X-based TE materials.^34,35^ Alloying Sn and Ge in Mg_2_Si can introduce point defects that induce short-wavelength photon scattering^23^ and mass difference scattering due to the large mass difference between the elements Si and Sn, thereby reducing thermal conductivity. For example, alloying with Sn has resulted in reported peak ZT-values of 1.4 for Mg_2_Si–Mg_2_Sn solid solutions, attributed to expanded valley degeneracy and decreased lattice thermal conductivity in the alloys.^36^ Additionally, adjusting the composition to achieve band convergence can lead to higher carrier mobility and power factor (S^2^σ). Complete band convergence has been reported in the range of *x* = 0.6–0.7 for Mg_2_Si_1–*x*_Sn_*x*_.^18^ Similarly, alloying with Ge in N-type Mg_2_Si_1–*x*_Ge_*x*_ solid solutions can improve ZT value to above 1.0, although slightly less effective than Sn addition.^23^ Various combinations of alloying elements (Si/Sn/Ge) with magnesium have been explored to enhance their TE properties as summarized in Figure 2a/b.^17^ Furthermore, other elements such as Cu, Ag, Ni, Zn, and In have been considered as acceptor dopants in Mg_2_Sn, albeit with limited success.^33^

Further doping of Bi^37^ and Sb^38^ into the Mg_2_X alloy system increased the maximum ZT values to 1.4 and 1.5, respectively, at 800 K.^36^ These advancements have spurred the development of another group of alloys based on Mg_3_Sb_2_/Mg_3_Bi_2_ Zintl compounds and MgAgSb. Mg_3_Sb_2_ compounds possess a typical cubic antibixbyite symmetry of the minerals, with dynamically stable *Ia*3̅ and *P*3̅*m*1 phases at ambient conditions.^31^ The *P*3̅*m*1 phase of Mg_3_Sb_2_ (Figure 1b) attracts much attention for its good TE performance. Mg_3_Bi_2_ shares the same crystal structure as Mg_3_Sb_2_, and forms a complete solid solution within the entire composition range.^39^ N-type Mg_3_Sb_2_ has demonstrated excellent TE performance due to much higher carrier mobility and larger band degeneracy, achieving a peak ZT of 1.5 in N-type Mg_3_Sb_2_-based materials at around 700 K through chemical doping, slightly excess Mg addition, and microstructure engineering.^40,41^ By alloying with Bi, the band gap of Mg_3_Sb_2-x_Bi_*x*_ is reduced, leading to excellent TE performance at near-room-temperature, enabling its application as a solid-state cooling alternative.^42^ Doping of Mn (Mg_3.2–*x*_Mn_*x*_Sb_1.5_Bi_0.49_Se_0.01_, *x* = 0.01) enhances the carrier concentration and mobility from 3.92 × 10^19^ cm^–3^ and 9.85 cm^2^V^–1^s^–1^ (Mn free), to 4.23 × 10^19^ cm^–3^ and 29.50 cm^2^V^–1^s^–1^, respectively.^43^ Meanwhile, a peak ZT value of 1.6 was obtained at 723 K with the Mn doping (*x* = 0.02). By incorporation metallic inclusions such as Nb or Ta into the Mg_3_(Sb,Bi)_2_-based matrix, the electrical conductivity was enhanced and the lattice thermal conductivity was reduced, leading to a record-high average ZT > 1.5 with a maximum value of 2.04 at 798 K.^44^ Other alloying elements like Y, Sc, Se, Te, Tm, and Nd have also been found to boost the ZT value to 1.8–1.9, especially at elevated temperatures, but are restricted by their scarcity.^23,45,46^ However, the single valence valley with low carrier mobility at the Brillouin center results in a low power factor of the hole-doped Mg_3_Sb_2_, making it a poor P-type TE material despite its low lattice thermal conductivity.^47^

Fortunately, the P-type α-MgAgSb exhibits phonon glass electron crystal behavior, and a peak ZT value of 1.2 has been obtained by enhancing the phase purity of the hole-doped sample.^48^ The complex lattice structure of α-MgAgSb consists of a distorted Mg–Sb rock salt lattice rotated by 45° along the *c* axis and silver atoms inside the polyhedrons as shown in Figure 1c.^32,49^ Due to the natural point defects and the disordered rock salt sublattice, α-MgAgSb inherently possesses low lattice thermal conductivity, ranging from 0.51 to 0.76 W·m^–1^·K^–1^ at room temperature. Native Ag vacancies are the main intrinsic point defects in α-MgAgSb, leading to significant strain fluctuations and mass differences in lattice, which enhance phonon scattering. A high peak ZT of 1.3 and an average ZT of 1.1 in the temperature range of 300–500 K have been achieved in α-MgAgSb.^50^ Chemical doping-induced point defects by Yb and other elements like Ni or Zn in α-MgAgSb can also produce more phonon scattering, further suppressing the lattice thermal conductivity and improving ZT to 1.4 as shown in Table 1.^51^ α-MgAgSb stables at temperatures <560 K, limiting its application to a narrow temperature range for special structures.^52,53^Table 1 summarizes the highest ZT values obtained for different Mg-based TE alloys and their relative temperatures. Zhou et al. compared the performance of Mg-based TE materials with the other recently developed TE materials such as Half-Heusler, Sn(Se.S) and tetradymites demonstrating its competitiveness in the midtemperature range.^23^

**Table 1:** Reported Highest ZT of Mg-Based Thermoelectric Materials

materials	composition	type	ZT_max_	temperature (K)	ref
Mg_2_Si	Mg_2_Si:Bi (1:0.02)	N	0.86	862	(55)
Mg_2_Sn	Mg_2_Sn	N	0.62	650	(17)
Mg_2_Ge	Bi doped Mg_2_Ge	N	0.32	750	(29)
	Li doped Mg_2_Ge	P	0.50	700	(56)
Mg–Si–Sn	Mg_2.15_Si_0.28_Sn_0.71_Sb_0.006_	N	1.3	700	(57)
	Mg_2.08_Si_0.364_Sn_0.6_Sb_0.036_	N	1.5	716	(58)
	Li doped Mg_2_Si_0.4_Sn_0.6_	P	0.6	700	(59)
Mg–Si–Ge	Mg_2_Si_0.6_-Ge_0.4_Bi_0.02_	N	1.0	900	(60)
Mg–Sn–Ge	Mg_2_Sn_0.75_Ge_0.25_	N	1.4	723	(61)
Mg–Si–Sn–Ge	Mg_2.16_(Si_0.3_Ge_0.05_Sn_0.65_)_0.98_Sb_0.02_	N	1.45	775	(38)
Mg_3_Sb_2_ or Mg_3_Bi_2_	Y-doped Mg_3.2_Sb_1.5_Bi_0.49_Se_0.01_	N	1.87	773	(62)
	Mg_3_Sb_1.5_Bi_0.49_Te_0.01_Nb_0.1_	N	2.04	798	(44)
	Mg_3.2_Nd_0.03_Sb_1.5_Bi_0.5_	N	1.8	725	(45,63)
MgAgSb	MgAg_0.97_Sb_0.99_	P	1.2	450	(48)
	Mg_0.995_Yb_0.005_Ag_0.97_Sb_0.99_	P	1.4	550	(51)
	MgAg_0.965_Ni_0.005_Sb_0.99_	P	1.4	450	(48)
	Mg_0.97_Zn_0.03_Ag_0.9_Sb_0.95_	P	1.4	423	(64,65)

Apart from the bulk materials development, the performance of the Mg-based coatings was also investigated. Mg_2_Si thin films were prepared by thermal evaporation of Mg and subsequent annealing at 623 K, which suppressed evaporation of Mg, decomposition of Mg_2_Si and oxidation of Mg_2_Si. A relatively high Seebeck coefficient of −235 μV·K^–1^ and a low thermal conductivity of 1.4–1.7 W·m^–1^·K^–1^ were obtained at 712 K for the polycrystalline Mg_2_Si thin film, resulting in a ZT of 0.68.^54^ Stochiometric Mg_2_Sn coatings, deposited by magnetron cosputtering of Mg an Sn with separated targets, achieved the best figure of merit, ZT = 0.27, at 473 K.^33^ Although the ZT value is generally lower than that of the bulk material, the lesser amount of material used provides them with an economic advantage.

## 3 Challenges and Major Issues of the Magnesium-Based TE Materials and Modules

Pursuing high ZT through the implementation of diverse phonon engineering and electron engineering schemes has been a focal point of the entire TE community. However, large electric fields, high thermal gradient, and elevated working temperature require high thermal stability to withstand temperature fluctuations and maintain materials composition, microstructure, and properties. There are a few barriers to bring these materials and technologies to market, such as inadequate mechanical properties, insufficient thermal stability, unreliable contacts, and the lack of matched P-type based materials.^66^

### 3.1 Mechanical Failure

Considering the operating conditions encountered during TE generation, stress is primarily induced by the large temperature difference between the hot and cold sides of the device. Additionally, the mismatch of coefficient of thermal expansion (CTE) between TE legs and contact materials would lead to extra stress. These create unique conditions for the deformation of TE legs and other elements of TE modules, which differ from those encountered under uniform heating. Furthermore, shock loads, vibration, and cyclic temperature effects in mobile applications can adversely affect the device’s integrity and stability.^12^ Thermomechanical properties are vital for the production and consistent functionality of TEG materials.

**Figure 3:**
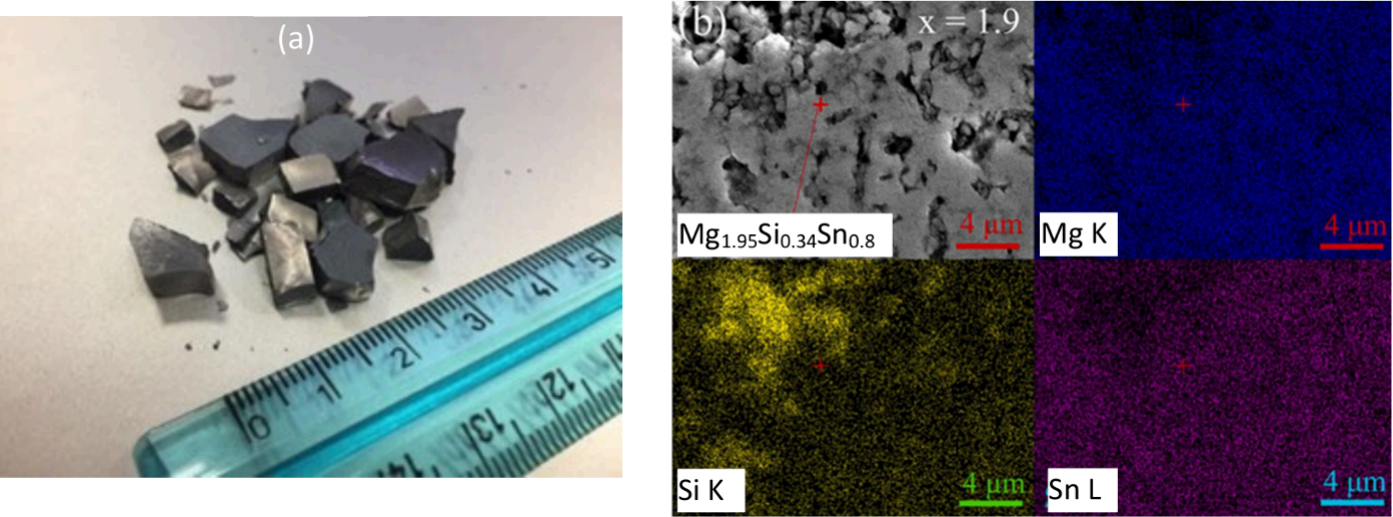
(a) Some of the broken Mg_2_Si_0.4_Sn_0.6_ pellets after sintering in our trial test, (b) EDS mapping on sintered magnesium deficient Mg_1.9_(Si,Sn) alloy.^70^ Reproduced with permission from ref (70), Copyright [2023] [Elsevier].

Inorganic TE semiconductor materials are brittle due to the crystalline structure, which contains intrinsic ionic, covalent, and/or van der Waals bonds, allowing easy cleavage along the ab-plane.^67^ As shown in Figure 3a, we found that the Mg_2_Si_0.4_Sn_0.6_ pellet were very easy to break after sintering if not controlled properly due to its low mechanical strength. TE materials are typically polycrystalline samples produced through melting or powder metallurgy methods, which inevitably contain a high concentration of defects or flaws, leading to reduced mechanical strength.^68^ The flaws limiting strength include volume type (e.g., pores/cavities, agglomerates, porous regions, inclusions, and large grains), surface type (e.g., handling damage, machining damage, pitting, oxidation, and chemical product), and edge type (e.g., edge chipping).^69^ As shown in Figure 3b, the Mg-deficient compound Mg_1.9_(SiSn) sintered at 973 K for 4 h in a vacuum after mechanical alloying had different defects like elemental agglomeration (Si), pores, etc.^70^ These defects lead to unpredictable changes or degradation in TE performance. Meanwhile, suitable mechanical strength and hardness are crucial to prevent surface damage during handling, and leg production such as the cutting and packing process. TE legs also require sufficient toughness to enhance productivity and prevent failure due to thermal fatigue or thermal shock during the repetitive heating and cooling cycles encountered in practical applications. The conventional TE device consists of bulk TE materials and electrodes through a rigid connection. During service, the accumulation of structural defects, external shear stress, and thermal stress can cause cracking, warpage, and the mechanical destruction of TE legs, contact structures, and other elements of modules, leading to a surge in internal resistance. Due to the limited compressive strength, the TE material may also fracture, leading to device failure.^69^

The elastic modulus of the Mg_2_Si-based TE materials ranges from 58 to 145 GPa, with their corresponding hardness values ranges from 2.4 to 5.6 GPa, which are higher than those of Bi_2_Te_3_ (32–52 GPa) and PbTe (27–58 GPa) based TE materials.^71,72^ These values are affected by differences in grain size according to the production methods, i.e., induction melted casting or spark plasma sintering. The Mg_2_Sn compound shows a Young’s modulus of 82 GPa and a hardness of 1.7 GPa.^72^ The formation of defects, such as pores and cracks can reduce the elastic modulus.^73^ The modulus reduces with increasing temperature and easily deforms above the yield stress at elevated temperatures. Mg_2_Si_1–*x*_Sn_*x*_ solid solutions exhibit a reasonable value of Vickers hardness (3.07–3.54 GPa) but demonstrate low fracture toughness in a range of 0.64–1.0 MPa·m^1/2^.^16,72,74^ This value falls between the lowest (0.35 MP·m^1/2^) for PbTe and the highest (2.8 MPa·m^1/2^) for Ca_3_Co_4_O_9_,^71^ and is comparable to the soda-lime glass (0.7–0.8 MP·m^1/2^).^75^ The bending strength of Sb-doped Mg_2_Si fabricated by SPS is 57 MPa at room temperature,^73^ and the tensile strength for Mg_2_Si with glass inclusions was ∼2 MPa.^76^

When TE modules are subjected to mechanical and thermal stresses, defects experience significantly higher stress compared to the average stress. This is because cracks propagate easily in brittle materials, even under low external loads. No noticeable plastic deformation occurs before fracture, and cracks develop due to thermomechanical stress when operating at high temperatures. Mg_2_Si-based TE materials are brittle with poor fracture toughness and flexure strength, so improving their toughness is a critical issue for practical usage in power generation.^77,78^

### 3.2 Chemical Stability of the Materials

Chemical stability is crucial for TE materials, and major issues include chemical interactions with other structural members, such as oxidation, decomposition, and sublimation of volatile element(s) which are significant hurdles for the application of these materials.^79−83^

#### 3.2.1 Oxidation

Magnesium and its alloys undergo catastrophic oxidation at temperatures above 673 K, and silicon begins to oxidize rapidly at a temperature of 973 K.^84^ The Mg_2_Si surface is highly reactive due to the existence of the Mg, resulting in a low standard Gibbs free energy of formation of MgO.^73^ Inoue et al. reported that Mg(OH)_2_ and MgH_2_ were formed on the Mg_2_Si powder surface in air at room temperature, followed by the formation of Mg(CO)_3_ layer with CO_2_ on Mg(OH)_2_ layer. Upon heating to 573 and 623 K, these compounds decompose, and Mg_2_Si starts to oxidize above 753 K (eq 1), with the Mg_2_SiO_3_ phase forming at 873 K (eq 2).^85^
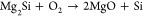
1
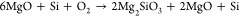
2

**Figure 4:**
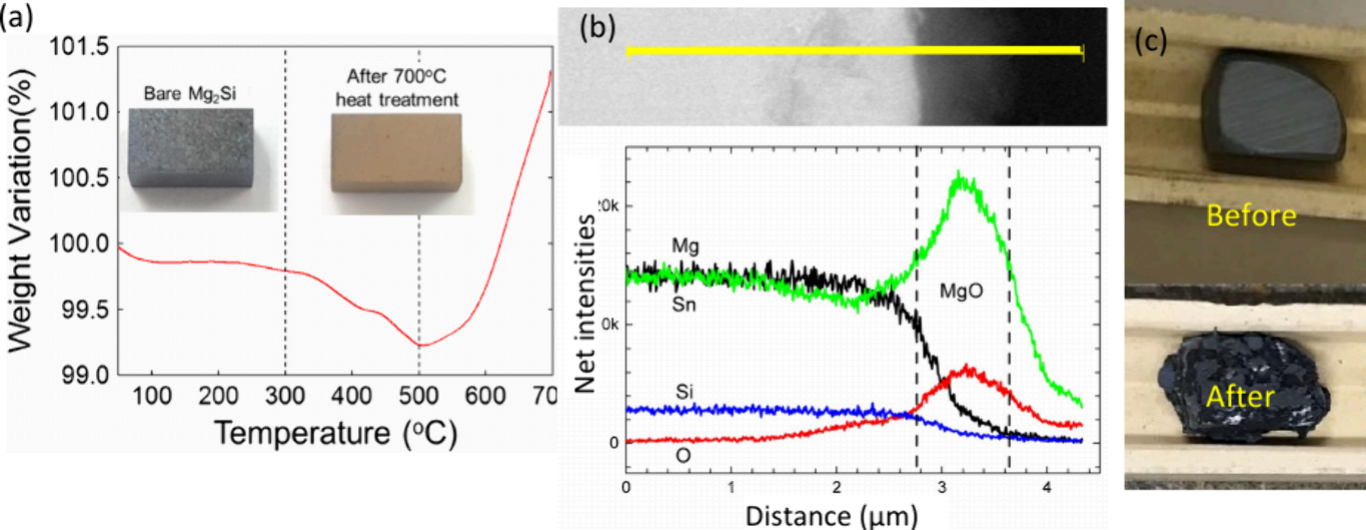
(a) TGA analysis of SPS sintered Mg_2_Si pellet, with inset pictures of Mg_2_Si pellets before and after heat treatment at 973 K for 1h.^86^ Adapted with permission from ref (86), Copyright [2016] [Elsevier]. (b) EDS line-scan of the 1 μm thick surface oxide layer on Mg_2_Si_0.4_Sn_0.6_Sb_0.01_ after 48 h in air at 673 K. A noticeable depletion of Mg directly beneath the oxide layer is clearly identified;^27^ Reproduced with permission from ref (27), Copyright [2016] [Elsevier]. (c) Mg_2_Si_0.888_Sn_0.1_Sb_0.012_ compound before and after treatment at 973 K/50h in our lab.

As shown in the TGA test of Mg_2_Si in the work of Park et al. (Figure 4a**)**, the weight slightly reduced from around 573 K due to the removal of residual moisture or the sublimation of elemental Mg, which has a relatively high vapor pressure.^86^ A significant weight gain appeared at about 773 K and increased sharply up to 973 K, leading to the gradual oxidation of the surfaces of the Mg_2_Si. The dark gray color of the Mg_2_Si pellet surface changed to dark yellow (inset picture in Figure 4a) after 1h exposure to air at 973 K.

Tani et al. reported that the Mg_2_Si reacted with O_2_ in the air above 723 K to yield MgO and Si, forming an 8 μm thick oxide layer on the surface after heat treatment at 873 K for 3 h.^87^ The oxidation is diffusion-controlled with an activation energy of 177 kJ/mol, calculated using test data between 773 to 923 K. Only MgO was formed on the evaporatively deposited Mg_2_Si film at lower temperatures, while SiO_2_ started to form when the temperature rose above 983 K, and MgSi_2_O_4_ was found in the oxide scale when the temperature reached 1213 K.^27^ The MgO layer on Mg_2_Si-based TE materials was a few nm thick under ambient conditions and increased to approximately 1 μm at elevated temperatures, providing protection up to temperatures around 723 K in air.^27,88^ The oxidation followed parabolic kinetics, and the oxide growth was controlled by outward Mg^2+^ diffusion through MgO.^27^ The onset (ignition) temperature of catastrophic oxidation for Mg_2_Si was about 1313 K.^27^

The oxidation of tin speeds up at 423 K, and Mg_2_Sn forms no passivating layer at high temperatures. The ignition temperature of the catastrophic oxidation for Mg_2_Sn is about 673 K, which is much lower than that for Mg_2_Si, and the oxide layer grows linearly with time.^27,89^ Adding Sn to Mg_2_Si can improve the TE performance, but it also makes the materials more susceptible to oxidation due to the low melting point of tin at about 505 K. The oxidation rate of the Mg_2_Si_1–*x*_Sn_*x*_ alloys (*x* = 0.1–0.6) was slow for temperatures below 703 K, but breakaway oxidation occurred at higher temperature ranges, and the onset temperature decreased with increasing levels of Sn in the alloy.^27,90^ Mg_2_Si_1–*x*_Sn_*x*_ powders started decomposing into MgO, Si and Sn at 630 K; whereas the dense pellets decomposed at a significantly slower rate compared to the powder samples due to their smaller specific surface area.^90^ In the meantime, the oxidation resulted in the redistribution of Mg (Figure 4b), which could compromise the performance of the TE. Further investigation suggested that a nonprotective MgO layer and the Sn-rich liquid at the interface led to the breakaway oxide layer.^27^ Mg_2_Si_0.4_Sn_0.6_ pellets oxidized easily and disintegrated into powders after heating at 823 K for 12 h in air.^91^ It also oxidized in an inert N_2_ gas atmosphere. Although no obvious structural change in the slightly oxidized Mg_2_Si_0.4_Sn_0.6_ sample at lower temperatures, the carrier concentration reduced clearly since oxidation created Mg vacancies in the lattice.^91^ We also found that the Mg_2_Si/Sn alloy pellet became dark, segregated, and distorted after being heated to 973 K for 50 h (Figure 4c). Mg_2_Ge also tends to absorb moisture from the atmosphere/humid air leading to its decomposition, which requires special care in storage.^92^

Mg_3_(Sb,Bi)_2_-based materials also exhibit weak stability, including Mg oxidization and loss, decomposition at high temperatures, as well as possible deliquescence in humid environments. The relative instability of the Mg^2+^ lattice (in octahedral site) can be attributed to the small ionic radius. Increasing the Bi content in Mg_3_Sb_2–x_Bi_*x*_ would weaken interlayer bonding and result in poorer thermal stability.^93^ Additionally, at elevated temperatures, the precipitation of the Sb/Bi phase and Mg loss can be observed, influenced by the high vapor pressure of Mg. MgAgSb is well-known to exist in three phases between 300 and 693 K. α-MgAgSb possesses a tetragonal structure with a distorted rock-salt lattice. and remains stable up to 573 K, demonstrating decent TE properties.^92^ However, secondary phases and impurities like Ag_3_Sb and pure antimony in the system often compromise performance. Intermediate temperature β-MgAgSb is stable up to 633 K, above which it transforms to a high-temperature phase (γ-MgAgSb).^49,52^ Both of these phases are not favorable for the TE function.^53^

#### 3.2.2 Sublimation and Dissociation

While heating a TE couple in an inert atmosphere can prevent oxidation, sublimation of species such as Sb from TE materials at elevated temperatures also causes performance degradation. Indeed, TE materials containing elements with high vapor pressure such as Pb, Ge, Te, Sb, Sn, etc., typically exhibit high sublimation rates at elevated temperatures.^71^ For instance, when heated to 773 K in a vacuum, the Mg_2_Si_0.3_Sn_0.7_ solid solution experiences significant Mg loss due to the high vapor pressure of Mg. Conversely, when heated in air, the sample oxidizes.^94^ For the Mg_2_(Si–Sn) materials, above a certain ignition temperature, the passivating outer layer of MgO breaks down, and oxidation proceeds exponentially due to the formation of liquid Sn below the MgO layer.^27^ The TE materials also exhibited decreased carrier mobility and carrier concentration due to the Mg loss and Sn precipitation during the heat treatment.

**Figure 5:**
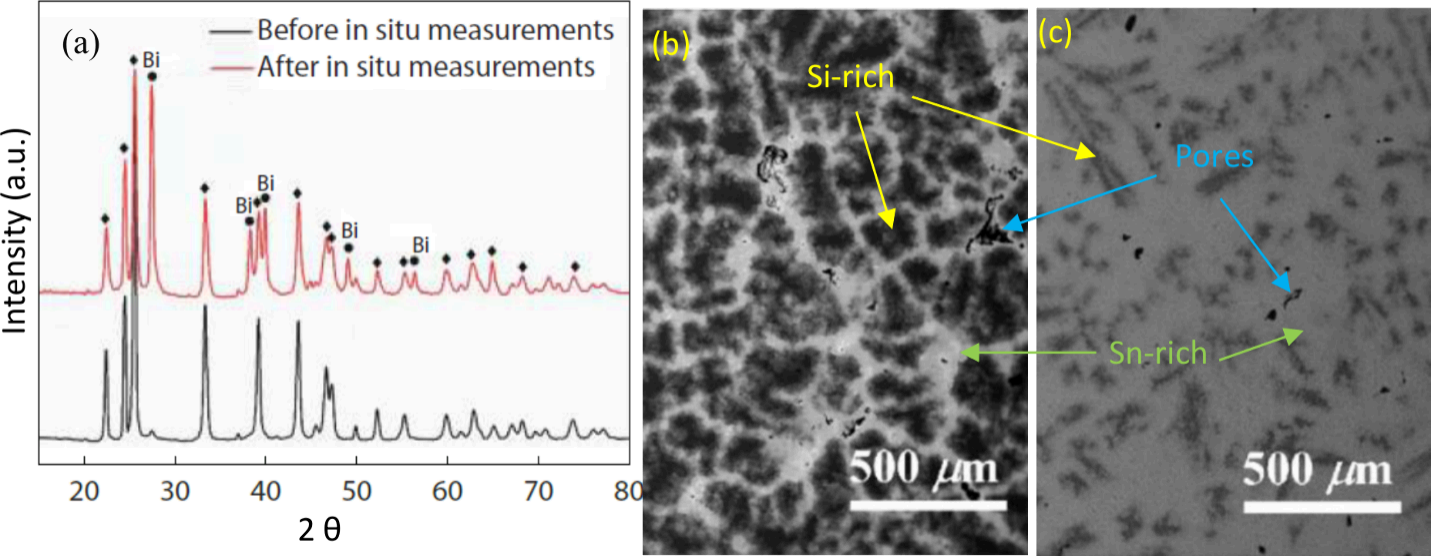
(a) Bismuth phase on the surface of the N-type Mg_3.2_Sb_0.49_Bi_1.5_Te_0.01_ after heating at 773 K,^96,97^ Reproduced with permission from ref (97), Copyright [2020] [Elsevier]. SEM-BE images display various amounts of dendritic microstructure in the two alloys: (b) Mg_2_Si_0.75_Sn_0.25_ and (c) Mg_2_Si_0.25_Sn_0.75_.^30^ Reproduced from ref (30), Copyright [2020] [American Chemical Society].

Mg_3_Sb_2–*x*_Bi_*x*_ alloys possess decent TE properties in the temperature range between 300 and 773 K; however, their thermal stability remains a problem. Approximately 11 wt % elemental bismuth in the N-type Mg_3_(Bi,Sb)_2_ crystallized as a secondary phase after the first heating cycle from 300 to 725 K, leading to the decomposition of the compound.^95^ Bismuth was released from the N-type Mg_3.2_Sb_0.49_Bi_1.5_Te_0.01_ crystal structure after heating at 773 K (Figure 5a).^96,97^ At a temperature of 773 K for 6 h, the average concentration of Mg in the Mg_3_Sb_2–*x*_Bi_*x*_ alloys changed from 63.61 at% to 52.43 at%. indicating a significant loss of Mg.^97^ At a vapor pressure of 10 Pa, the corresponding temperatures for Mg, Sb, and Bi are 773, 876, and 1041 K, respectively. The low melting temperature of Mg accounts for its loss at this temperature. Meanwhile, Sb loss was also observed.^97^

#### 3.2.3 Materials Miscibility

Mg_2_Si_1–*x*_Sn_*x*_ materials have decent TE performance within the intermediate temperature range (300–773 K), heavily influenced by the ratio of Si to Sn. Above 773 K, Mg_2_(Si,Sn) material system suffers from Mg loss. However, Mg_2_Si and Mg_2_Sn are not completely miscible, and an immiscibility region typically occurs for x values between 0.4 and 0.6.^94^ It is commonly reported that within the miscibility gap, phase separation occurs followed by the formation of elemental Si and Sn, or an Sn–Mg melt due to the loss of Mg from Mg_2_(Si,Sn).^98^ Due to the miscibility gap, separation into Sn-rich and Si-rich phases could occur in Mg_2_(Si,Sn) during heating and cooling, leading to expansion and porosity resulting from Kirkendall effect in the materials, as displayed in Figure 5b.^30,99^ These processes are detrimental to Mg_2_(Si,Sn) because they signify low thermal stability of the material system, primarily due to the loss of Mg.

### 3.3 Unreliable Contact and High Contact Resistance

Direct soldering of most TE materials poses challenges due to either poor wettability of solder on TE materials or subsequent reaction/diffusion between the materials at elevated temperatures. Other issues include diffusion and self-diffusion in TE materials and contact structures, as well as the formation of intermetallic compounds in contact structures, resulting in unreliable contact between the TE leg and the electrode.^100^ The choice of different electrode materials further complicates the production of the TE module, including electrode fabrication, interface optimization, and protective coating. The reliability and compatibility of the metallized contact layer on both TE materials pose a significant challenge for constructing TE modules.

**Figure 6:**
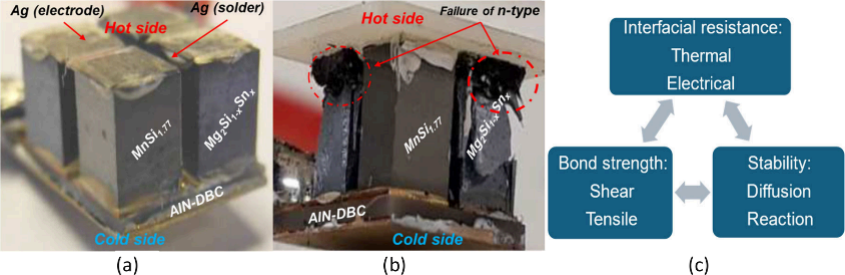
TE generator (a) before and (b) after thermal cycling test for 100 h (423–673 K);^99,102^ reproduced from ref (102), Copyright [2024] [American Chemical Society]. (c) Challenges to the TE contact interface.^101^

Degradation of contact structures primarily occurs due to interfacial reactions at the junction between the contact material and the semiconductor materials of the TE leg at high temperatures. For example, the electrical resistance between the Mg_2_Si TE leg and the Cu electrodes was around 0.7 Ω after production; however, after 1 h of treatment at 973 K, the contact resistance increased to almost 18 Ω which was a 25-fold increase, mainly originating from the oxidation of the TE leg.^86^ The formation of intermetallic compounds due to these reactions markedly raises the electrical resistivity of the contacts. These intermetallic compounds, being brittle, develop a porous structure over time when subjected to temperature gradients, which leads to cracking. This not only increases electrical contact resistance but also causes the breakdown of contact structures. In the case of soldered contacts, the solder contents can penetrate the TE material, compromising its performance. Another issue is the diffusion of elements in TE materials and the contact materials, which can lead to the formation of precipitates, as well as the creation of structural defects, especially dislocations. Diffuse degradation resulted in the deteriorating TE properties and a reduction of their mechanical strength. As shown in Figure 6, a TEG comprising two legs of high manganese silicide (HMS) and two legs of Mg_2_Si_0.55_Sn_0.45_ was assembled by soldering the TE legs to metallized ceramic plates (AlN-DBC) using silver solder. A thin layer of Au/Ti (300/100 nm) served as a diffusion barrier between the (N & P)-type and the solder. After 100 h (400 cycles) of operating, fractures (cracks) were localized on the hot side of the Mg_2_Si_0.55_Sn_0.45_ legs. During thermal testing, the internal electric resistance of the TEG increased, likely due to the formation of a thin layer of MgO and a diffusion layer between the TE leg and the solder (Ag) on the hot side of the legs.^99^

In additions to the properties of TE materials, the practical energy conversion efficiency and service life of TE devices are highly determined by the assembling process and the contact interface. Liu summarized the challenges and the interrelationship among different requirements and designs for the interface contact, as shown in Figure 6c.^101^ Therefore it is crucial to achieve a reliable contact through special pretreatment of the semiconductor surface with desirable roughness, the creation of an anti-diffusion/barrier layer, and the application of a sublayer/adhesion layer that enhances the bond strength of the contact structure with the TE material.^66^

## 4 Enhancements of the Performance of the TE Materials/Modules

To address the degradation of TE materials and modules, various approaches have been attempted to protect them and enhance their performance and durability. In this section, we will address the main points regarding strengthening mechanical strength, protecting the TE module from degradation, and improving contact for better performance of TE modules.

### 4.1 Strengthening Mechanical Strength

While the toughness of silicide-based TE materials is relatively higher than that of other TE materials, it remains as brittle as glass. Therefore, enhancing fracture toughness is essential to prevent or deflect propagating cracks induced by external loads and thermal stress.

#### 4.1.1 Grain Refinement

Grain refinement is an effective technique for enhancing the toughness and strength of brittle polycrystalline materials: the smaller the grain size, the stronger the mechanical properties become. Nanostructuring has been found to have a beneficial effect on the physicochemical and mechanical properties of the Mg_2_Si–Mg_2_Sn solid solutions, resulting in a substantial increase in mechanical strength and resistance to oxidation. However, further decreasing the grain size to the nanoscale does not influence the TE efficiency in the operating temperature range.^34^

Conventional milling techniques such as planetary and vibratory ball milling have been utilized to reduce grain size. Schmidt et al. examined Mg_2_Si processed by powder metallurgy and sintered via pulsed electrical current sintering.^103^ As the mean grain size reduced from 3.9 to 2.4 μm, the Vickers hardness and fracture toughness increased from HV_1.0_5.0 GPa and K_IC(9.8N)_ of 0.9 MPa·m^1/2^ to HV_1.0_5.4 GPa and K_IC(9.8N)_ of 1.3 MPa·m^1/2^. Wang prepared nanocrystalline Mg_2_Si intermetallics (d ≈ 54 nm) using mechanically activated solid-state reaction plus hot-pressing, and its toughness reached 1.67 MPa·m^1/2^, which is the highest value reported in the literature.^104^ The nanostructured MgAgSb TE materials with a grain size of about 150 nm, prepared by ball milling and hot press process, exhibited significantly strong mechanical properties. As shown in Table 2, Young’s modulus, nanoindentation hardness, compressive strength, and fracture toughness are 55.0 GPa, 3.3 GPa, 389.6 MPa, and 1.1 MPa·m^1/2^ respectively.^105^

**Table 2:** Mechanical Properties of Selected Mg-Based TE Materials

materials	hardness (GPa)	compressive strength (MPa)	flexural strength (MPa)	fracture toughness (MPa·m^1/2^)	Young’s modulus (GPa)	ref
Mg_2_Si	4.8–5.6			0.7–1.67	117	(103,104)
Sb-doped polycrystalline Mg_2_Si		430.5	55.4	0.62	105	(106)
SiC reinforced Mg_2_Si	4.8			0.50–1.30	112	(107)
Mg_2_Si_1–*x*_Sn_*x*_ (*x* = 0.4–0.6)	3.5	492	79	0.99	83	(108)
Mg_2_Si_1–*x*_Sn_*x*_	2.7	458	72	0.91		(16)
SiC reinforced Mg_2_Si_1–*x*_Sn_*x*_	2.8–3.0	490–599	71.3–84.5	1.17–1.36		(16)
Mg_2_(Si_0.3_Sn_0.7_)_0.99_Sb_0.01_			73	0.62		(109,110)
GO/CNTs reinforced Mg_2_(Si_0.3_Sn_0.7_)_0.99_Sb_0.01_			90	0.90		(110)
α-MgAgSb	3.3	390		1.10	55	(105)
2%ZrO_2_ doped Mg_3.2_Sb_1.99_Te_0.01_		565–669				(111)

Theoretical analyses have suggested that grain refinement becomes effective when the grain size is reduced to less than 100 nm. However, it noted that as the grain diameter decreases, oxidation becomes more prevalent due to the significantly larger specific surfaces and grain boundaries in fine grains. Producing bulk materials with nanosized grains is challenging because surface oxidation can lead to impurity formation, particularly MgO.^112^ De Boor et al. found that even a small amount (7 wt %) of MgO can result in a significant reduction (30%) in ZT. Thus, they recommended optimizing processing parameters such as grain size, process temperature, and sintering temperature to fabricate pure Mg_2_Si without impurities.

#### 4.1.2 Addition of Second Phases

The addition of second phases such as fibers, whiskers, flakes, and particles has proven effective in enhancing toughness. For particulate composites with a volume fraction of ∼10%, toughness can reach up to 2.2 MPa·m^1/2^.^73^ Nanophases with high fracture toughness, such as SiC, CNTs (carbon nanotubes), or graphene/graphene oxides, have been extensively investigated for their potential in improving the fracture toughness of TE materials.^78^

Schmidt et al. discovered that incorporating ∼2 vol % SiC nanoparticles via a planetary ball mill enhanced the fracture toughness of Mg_2_Si by a third, while the Young’s modulus (∼112 GPa) and hardness (∼4.8 GPa) remained relatively insensitive to the addition of 0–4 vol % SiC nanoparticles.^107^ This improvement in fracture toughness and the compressive strength was attributed to the pinning effect, fiber bridging, and fiber pull-out mechanisms in the Mg_2.16_(Si_0.3_Sn_0.7_)_0.98_Sb_0.02_ composite with 0.8 at% SiC nanopowders or nanowires, resulting in enhancements of about 50% and 30%, respectively (Table 2). A maximum ZT value of 1.2–1.3 was achieved at 750 K.^16^ Moreover, the flexural strength and Vickers hardness of the composites at room temperature were also enhanced to various degrees. Inoue et al. introduced 10 vol % SiC into the Mg_2_Si grains using a plasma-activated sintering process, resulting in a toughness of 1.02 MPa·m^1/2^ for the intragranular Mg_2_Si/SiC composite, which was 60% higher than that of pure Mg_2_Si, with only a small reduction in electrical conductivity.^106^

The incorporation of a small quantity (0.25–1 vol %) of conductive glass-frit leads to a notable enhancement in the mechanical properties of the mechanically alloyed and hot-pressed Mg_2_Si by eradicating microcracks inherent in the brittle Mg_2_Si system.^76^ Al-doped samples containing conductive glass-frit achieved a power factor times temperature (S^2^σT) of >2 W·m^–1^·K^–1^.

**Figure 7:**
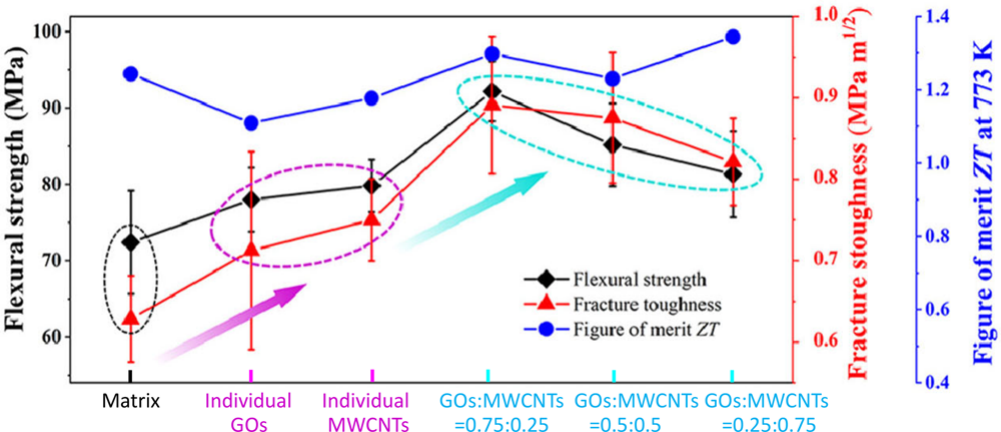
Mechanical and thermoelectric properties of Mg_2_(Si_0.3_Sn_0.7_)_0.99_Sb_0.01_ reinforced by GO nanosheets and multiwalled CNTs.^110^ Reproduced with permission from ref (110), Copyright [2021] [Elsevier].

As demonstrated in Figure 7, different concentrations of graphene oxide nanosheets (GOs) and multiwalled carbon nanotubes (MWCNs) were hot pressed together with Mg_2_(Si_0.3_Sn_0.7_)_0.99_Sb_0.01_ powders to fabricate TE composites. A significant improvement, with a 27% in flexural strength and a 41% in fracture toughness through crack bridging, was achieved without compromising the TE properties when 75%GOs/25%MWCNs were added.^110^ However, careful consideration is needed when incorporating nanoparticles as they can potentially reduce TE performance.^113^ By simultaneously activating three different inhibition mechanisms for crack propagation—bridging of cracks, sheet pullout within the crack, and deflection of crack propagation—the incorporation of dual nanoinclusions of reduced graphene oxides (rGOs) and Sn NPs (50–150 nm) into Al and Bi codoped Mg_2_Si TE materials enhanced fracture toughness to 2.26 MPa·m^1/2^ from 0.82 MPa·m^1/2^ for pristine Mg_1.96_Al_0.04_Si_0.97_Bi_0.03_. However, the TE performance declined due to the deterioration of electronic transport properties stemming from enhanced electron scattering.^113^

The addition of ZrO_2_ microparticles into Mg_3.2_Sb_1.99_Te_0.01_ increased the compressive and bending strengths to 669 and 269 MPa, respectively, from 565 and 193 MPa.^111^ Furthermore, the combined secondary phase reduced lattice thermal conductivity and increased electrical conductivity, albeit with a slight degradation in the Seebeck coefficient. The average ZT in the temperature range of 300 to 500 K reached 0.8.

#### 4.1.3 Summary

The use of nontraditional processing technologies, such as pressure-induced sintering, can strengthen the fracture toughness; however, its effect is limited to the intrinsic property of the materials. Grain refinement is an effective method to reinforce the toughness, but the process must be optimized to avoid excess oxidation caused by the increased specific area. The addition of second phases attracts much interest due to its significant impact, but care must be taken to avoid adversely influencing the thermoelectric properties of the materials.

### 4.2 Protection of the TE Legs or Modules

#### 4.2.1 General Methods of Protection

The major factors affecting the stability of TE materials are the atmosphere and elevated temperature. One solution is to seal the TEG devices with an inert gas like argon or to operate in a vacuum, which reduces oxidation. Kambe et al. encapsulated the SiGe or BiTe TE modules in a vacuum-tight stainless-steel container to prevent oxidation at intermediate temperatures (from 573 to 973 K) during power generation.^114^ Salvador et al. encapsulated the Skutterudite-based TE modules in aerogels via the Sol–Gel method by casting an ortho-organo-silicon-based sol mixture and catalyzing a condensation reaction to form a silicon oxide gel.^115^ However, the significantly rising costs of these metallic housing structures and the additional reliability issues of long-term sealing make this technology difficult to apply. Meanwhile, the sublimation of volatile alloying components and impurities occurs most extensively in a vacuum, increasing with the temperature. Safety issues arise from using inert gas to protect the TE modules, generating extra cost and making the system more complex, thus hindering its employment.

From a materials design point of view, oxidizing-resistant materials can be developed by modifying the fabrication route to improve the microstructure, thereby increasing the stability of the materials without the need for protective environments.^66^ For example, Mg_2_Si prepared by an all-molten method with no residual metallic-Mg exhibited atmospheric durability at 873 K for 1000 h.^73^ Others suggested that increasing the nominal content of Mg can improve thermal stability but it can lead to higher thermal conductivity and lower oxidation resistance.^116^ Due to the inherent characteristics of Mg-based TE materials, approaches to creating a barrier to volatile impurities and components from TE legs through surface coatings become more attractive.^117−119^ Due to the harsh working conditions, protective coatings for high-temperature applications should incorporate chemical stability, wetting properties, and thermal expansion compatibility. They should have good adhesion to TE materials and serve as an effective diffusion barrier against gases and/or liquids. Additionally, they should maintain stability when in contact with chemical agents.^120^

The most common coatings for high-temperature TE applications include oxides, nitrides, silicides, borides, carbides, or their mixtures.^121^ Coatings like Si_3_N_4_, CrSi, Al_2_O_3_, ZrO_2_, NbN, WN, TaN, MgAlO_4_, Enamel, Nano-SiO_2_, Mo/SiO_*x*_ multilayer, composite glass, aerogel, and heat resistance paint have been used to protect Skutterudites TE legs.^117−119,122,123^ Silica-based glass and glass–ceramic coatings have been used to protect PbTe up to 773 K,^124^ MnSi up to 873 K^125^ and Bi_2_Te_3_-based TE materials as well.^126^ DC magnetron sputtering (MS) AlTiN up to 2.6 μm can protect the Ni–Zn tetrahedrite well up to 723 K.^127^ However, there are limited reports of the coatings applied to Mg-based TE materials or modules. Table 3 demonstrates some research on improving the oxidation resistance and the thermal stability of Mg-based TE materials.

**Table 3:** Typical TE Material and Related Protection Coatings

TE materials	coatings and deposition methods	test	ref
Mg_2_Si	SiO_2_ coating via dip (Sol–Gel) coating	823 K, 200 h	(128,129)
Mg_2_Si_0.487_Sn_0.5_Sb_0.013_	silica based glass coating (slurry pasted and then fired at 823 K/1 h)	773 K, 120 h	(120,130)
Mg_2_Si	dip coated (Sol–Gel) black glass (SiOC)	up to 723 K	(131)
Mg_2_Si_0.4_Sn_0.6_	atomic-layer-deposited 18 nm Al_2_O_3_ film	823 K, 12 h	(91)
Mg_2_Si	dip coated with coating agent composed of SiO_2_, ZrO_2_, and mica	873 K, 7000 h	(73)
Mg_2_Si	plasma sprayed 50 μm thick 8 mol % yttria-stabilize zirconia (YSZ)	973 K, 1 h	(86)
		873 K, 10 cycles	
Mg_2_Si	RF MS multilayered MoSi_2_ film (2–3 μm)	873 K	(132)
Mg_2_Si	RF MS multilayered β-FeSi_2_ film (0.7 μm)	873 K, 3h	(87)
Mg_2_Si_0.888_Sn_0.1_Sb_0.012_	DC MS CrSi coating (3–5 μm)	773 K/1 h, 50 cycles	
Mg_2_Si_0.3_Sn_0.7_	spraying 0.5 mm BN coating	773 K	(94)
Mg_2.1_Si_0.487_Sn_0.5_Sb_0.13_	brushed and cured solvent-based resin	773 K, 120 h	(133)
Mg_3_Sb_2–*x*_Bi_*x*_	spraying and dried BN coating	>673 K	(97)
Mg_3_Sb_1.5_Bi_0.5_	Mg–Mn alloy coating	673 K, 30 days	(134)

#### 4.2.2 Oxide Coatings

Al_2_O_3_ thin film is a popular passivation material for silicon in the photovoltaic and microelectronic industries. Zhang et al. deposited a nanoscale amorphous Al_2_O_3_ coating by ALD.^91^ The dense, well-adherent and uniform Al_2_O_3_-coated Mg_2_Si_1–*x*_Sn_*x*_ pellets remained stable in inert gases at 823 K for 12 h, while the unprotected sample decomposed completely into MgO, Si and Sn under the same operating conditions. Surface passivation coating, aimed at blocking oxygen transmission, was one method used to deactivate the surface of Mg_2_Si. Initially, the Mg_2_Si surface was stabilized by forming a 10-μm thick surface passivation layer using an alkaline conditioner. Subsequently, the surface was dip-coated with a coating agent consisting of SiO_2_, ZrO_2_, and mica.^73^ After aging at 873 K for 7000 h in air, the Mg_2_Si TE chips remained durable with stable resistivity at 3.45–3.73 × 10^–6^ Ω·m.

The Mg_2_Si samples were coated with 9 μm SiO_2_ layer using a modified Sol–Gel route via dip-coating, followed by drying at room temperature and annealing in a vacuum for 1 h at 573 K. The silica-coated Mg_2_Si displayed excellent structural and TE properties, maintaining stability for up to 200 h at 823 K in air. In contrast, the surface of the uncoated pellets degraded, forming cracks after 30 h and crumbled after 200 h of aging.^129^

**Figure 8:**
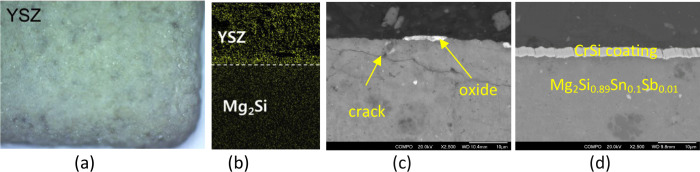
(a) SEM image of YSZ coated Mg_2_Si TE leg surface and (b) EDS oxygen distribution mapping after 10 thermal cycles between room temperature and 873 K;^86^ adapted with permission from ref (86), Copyright [2016] [Elsevier]. Thermal stability test for N-type Mg_2_Si_0.888_Sn_0.1_Sb_0.012_ heating to 773 K for 1 h: (c) 10 cycles for as received Mg_2_Si_0.888_Sn_0.1_Sb_0.012_ pellet, and (d) 50 cycles for PVD CrSi coated Mg_2_Si_0.888_Sn_0.1_Sb_0.012_ pellet (our research).

Furthermore, Park et al. tested different oxide coatings such as plasma sprayed alumina (Al_2_O_3_), yttria (Y_2_O_3_), 8 mol % yttria-stabilized zirconia ((Y_2_O_3_)_0.08_(ZrO_2_)_0.92_, YSZ), and plasma-nano coating deposited 200 nm of the 20 mol % samaria-doped ceria (Sm_0.2_Ce_0.8_O_1.9_, SDC). They found that the different CTE led to the cracks in the 50 μm thick alumina and yttria coating after heating to 973 K for 1 h. After this treatment, some local delamination occurred in the thin SDC coating, while the YSZ remained intact. YSZ exhibited excellent oxidation suppression characteristics for Mg_2_Si after 10 thermal cycles from room temperature to 873 K (Figure 8a,b).^86^ Coated Mg_2_Si TE leg with YSZ was also effective in stabilizing the contact and the electrode, as the contact resistance remained low at 0.7 Ω after heat treatment.^86^

A glass coating, containing a mixture of oxides including SiO_2_, K_2_O, Na_2_O, CaO, MgO, Al_2_O_3_, B_2_O_3_, was applied onto pellets of Mg_2_Si_0.487_Sn_0.5_Sb_0.013_, and fired at 823 K for 1 h.^130^ The glass coating exhibited a CTE of approximately 17 × 10^–6^ K^–1^, slightly lower than that of the substrate (17.6 × 10^–6^ K^–1^), resulting in a moderate compression state in the coating. This characteristic potentially enhanced the resistance of the coated Sb-doped Mg_2_(Si,Sn) to crack propagation during cooling process or thermal cycling. Following an aging test at 773 K for 120 h in air, the uncoated Mg_2_Si_0.487_Sn_0.5_Sb_0.013_ sample underwent complete oxidation, transforming into a mixture of powders including MgO, SnO_2_, SnO, Sn and Si, whereas the glass coated sample appeared unaffected. Nevertheless, further investigations regarding interface alterations and TE properties are necessary before application.^120^

Silicon-oxycarbide (SiOC), often referred to black glass, denotes a carbon-containing silicate glass in which oxygen and carbon atoms form bonds with silicon within an amorphous network structure. This materials boasts excellent mechanical properties and chemical stability.^135^ Magnesium silicide samples were covered with black glass (SiOC) amorphous coatings using dip-coating (sol–gel) method, followed by annealing in Ar gas atmospheres at various temperatures between 673 and 823 K. The protective coating exhibited continuity and good adhesion up to 723 K. However, at higher temperatures, it began to crack due to differences in CTEs between the coating and TE material.^131^

#### 4.2.3 Silicide and Similar Compounds

MoSi_2_ has a high melting point (2303 K), along with excellent oxidation resistance and diffusion barrier characteristics. Consequently, it has found application in high-temperature structural materials such as combustion chamber components, heating elements in oxidizing environments, and diffusion barriers in microelectronic devices.^136^ A 2.5 μm thick MoSi_2_ thin film barrier, deposited via RF magnetron sputtering, demonstrated good thermo-mechanical compatibility with the sintered Mg_2_Si pellet substrate. It efficiently protected the substrate up to 873 K. To maintain the Seebeck potential of the pellets, an insulating nanocomposite MoO_3_/SiO_2_ film was utilized between the substrate and the MoSi_2_ protective layer, aiming to reduce interference from the conductive coating. However, considerations regarding CTE mismatch and compositional instability arose at temperatures exceeding 773 K.^132^ β-FeSi_2_, an environment-friendly silicide semiconductor with an orthorhombic structure, exhibited remarkable oxidation resistance in the high-temperature region below 1073 K.^137^ RF magnetron sputtered β-FeSi_2_ on Mg_2_Si improved oxidation resistance up to 873 K.^87^ Upon heat treatment in air at 873 K for 3 h, an 8-μm thick oxide layer formed on uncoated Mg_2_Si samples. Conversely, Mg_2_Si samples coated with 0.7 μm thick β-FeSi_2_ films showed no oxide layer formation.

Recently, we investigated the thermal stability of the compact and uniform CrSi_*x*_ coatings deposited using a close field unbalanced magnetron sputtering technique applied to hot-pressed sintered Mg_2_Si_0.888_Sn_0.1_Sb_0.012_ TE pellets. The uncoated pellet oxidized and developed cracks on the surface region after 10 cycles of heat treatment at 773 K for 1 h (Figure 8c). In contrast, the CrSi-coated pellet remained intact and exhibited integrity after 50 cycles of test, indicating excellent stability (Figure 8d).

#### 4.2.4 Other Compounds

Boron nitride (BN) coating, applied to Mg_2_Si_0.3_Sn_0.7_ pellets by spraying a 0.5 mm layer of BN, provided effective protection up to 773 K. However, at 823 K, the carrier density in the sample deteriorated.^94^ Mg_3_Sb_2–*x*_Bi_*x*_ alloys tend to become unstable at temperature above 673 K, experiencing significant Mg loss and altered microstructures. Coating Mg_3_Sb_2–*x*_Bi_*x*_ alloys with BN effectively suppressed Mg loss, greatly enhancing their thermal stability.^97^ A commercial Mg–Mn alloy (ME20M) was used as protective coating layer for Mg_3_Sb_1.5_Bi_0.5_ TE leg. The dense and continuous ME20M-coated Mg_3_Sb_1.5_Bi_0.5_ sample remained stable for nearly 30 days at 673 K, which could effectively obstruct Mg escape and prevent oxygen penetration.^134^

A solvent-based resin (CP4040-S1, ARAMCO Scientific Company, Los Angeles, U.S.A.) was brushed onto Mg-based TE pellets and cured for 45 min at 523 K. After aging in air at 773 K for 120 h, the uncoated Mg_2.1_Si_0.487_Sn_0.5_Sb_0.13_ completely burned, turning into a powder consisting of various compounds (MgO, SiO, SnO_2_, and Sn). In contrast, the resin coated pellet, with a thickness of 30–100 μm, did not experience significant oxidation and remained mainly composed of a single phase, although some cracks and small amount of MgO were present due to imperfections in the coating.^133^

#### 4.2.5 Coating Selection Principles and Summary

In short, the selections of coating should adhere to the following principles:^120^

Low thermal conductivity to minimize parasitic heat loss.
Low electrical conductivity to prevent short-circuiting.
Coefficient(s) of thermal expansion matching that of the TE material, ensuring good interface compatibility and stress resistance during thermal cycling.
Sufficient thermal stability and mechanical strength for long-term durability.
No adverse impact on TE performance.

Oxides like silica/glass, silicides, and nitrides are the most used, and production methods include dip coating, Sol–Gel, plasma spraying, liquid spraying, atomic-layer deposition (ALD), and PVD etc. Silicon-based oxides and silicide are preferred coatings for the Mg_2_Si-based TE modules. While magnetron sputtering offers high-quality coatings, it comes with a higher cost. Plasma spraying, dip coating, and Sol–Gel techniques are more economical alternatives.

### 4.3 Improving the Contacts

TE module degrades over long-term, especially on the hot side, where interdiffusion and material loss are more prevalent. Metallization of the TE leg or collecting plate is of great importance by introducing functional layers, including a diffusion barrier layer, a contact layer, an adhesion layer and a compliant layer. The metallized layer assists the soldering process or reduces mutual diffusion. Due to the harsh working conditions, the ideal contact material should exhibit the following characteristics:^1,138^ high electrical conductivity and good thermal conductivity, CTE matching with the TE elements, capability to be made very thin to minimize total electrical and thermal resistances, low contact resistance at the interface between the contact layer and the TE surface, stability at elevated operating temperature, ability to form strong mechanical bonds with the TE layer, higher yield strength than solder at working temperature, and the electrode should have a melting point 20% to 50% higher than the joining temperature.^139^

The linear coefficient of thermal expansion (CTE) for Mg_2_Si can be expressed in the form:^140^
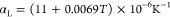
3Mg_2_(Si,Sn) based materials have a slightly higher CTE of 16.5–18.5 × 10^–6^ K^–1^.^130,141^

As shown in Table 4, metals like Cu, Ni, Ag, Al, Mo, Ti, Au, Pd, and their alloys are potential candidates for bonding with Mg-based TE materials, due to their low-resistivity and higher bond strength (4.3–17.3 MPa). Lower interfacial resistance of multielement alloys, such as (Co, Cr, Ti)Si_2_, Ni_45_Cu_55_, 304 stainless steel (304SS), and Mg_2_SiNi_3_, have also been reported.^142^

**Table 4:** Comparison of the Mg-Based TE Materials and the Metallic Electrodes^32,73,143−147^

	Mg_2_Si	Mg_2_Si_*x*_Sn_1–*x*_	MgAgSb	Mg_3_(Sb_1–*x*_Bi_*x*_)_2_	Cu	Ni	Ag	Al	Mo	Ti	Au	Pd
CTE (×10^–6^/K)	15.4	16.5–18.5	19–22	18.7–24	16.5	13.4	18.9	30.2	5.2	8.6	14.2	5.9
melting point (K)	1375	1051–1375	>793	1094–1500	1358	1728	1234	933	2893	1941	1338	1827
resistivity (10^–8^ Ωm)	250	400–500	1900–2500	1500–3000	1.7	7.04	1.6	5.92	5.6	43.1	2.2	10.6

#### 4.3.1 Copper Contact

Copper has a CTE very close to that of Mg_2_Si-based TE material. Ferrario et al. compared the metals (Cu, Ni, and Au) contacts deposited by DC magnetron sputtering onto Mg_2_Si samples.^148^ The adhesion of the sputtering deposited Cu film electrodes with a postannealing treatment showed the best adhesion performance and lower contact resistance than that of the Ni and Au layers. A 300-μm Cu sheet brazed on the N-type Mg_2_Si pellet by Ag_56_Cu_22_Zn_17_Sn_5_ alloy demonstrated the lowest contact resistances of 4.43 × 10^–5^ Ω·cm^2^ compared to the sputter-deposited contact layer.^148^ Cu electrodes were bonded to Mg_2_Si using the spark plasma sintering (SPS), which formed a 10-μm thick intermediate layer with Mg, Si, and Cu diffused into each other. The Mg_2_Si/Cu joints remained intact after annealing at 773 K for 72 h in vacuum, but the contact resistance increased with cracks developing along the boundary after aging at higher temperatures (823 K and 853 K).^149^

Cu foil can be joined with N-and P-type Mg_2_Si_0.3_Sn_0.7_ legs through direct or indirect resistive heating. Cu diffused into TE materials, creating relatively thick (200 and 100 μm, respectively) and complex reaction layers under both conditions. Electrical contact resistance remained less than 1 × 10^–5^ Ω·cm^2^ even after annealing.^100^ Ayachi et al. joined the Cu and Ni_45_Cu_55_ contacting electrodes to Mg_2_Si_0.3_Sn_0.7_ pellets by hot pressing in a current-assisted press.^150^ They found that Ni_45_Cu_55_ joining showed relatively low contact resistance of ∼3 × 10^–5^ Ω·cm^2^ but had a less inhomogeneous reaction layer, while Cu joins had much lower specific electrical contact resistance for both N- and P-type silicides (<1 × 10^–5^ Ω·cm^2^) with a wide, highly conductive diffusion regions. After annealing at 723 K for 1 week, the resistance values of the Cu joint increased up to ∼1 × 10^–4^ Ω·cm^2^ for annealed N-type samples but remained low (<1 × 10^–5^ Ω·cm^2^) for P-type.^150^ Cu can diffuse into the TE materials and react with them, causing a negative effect on the TE performance and occasionally leading to local delamination of the electrode.^151^ To reduce Cu diffusion during sintering, SS 304 interlayer was used between Mg_2_Si_0.4_Sn_0.6_/Cu contacts.^152^ Without the SS 304 layer, the electrical resistivity of Mg_2_Si_0.4_Sn_0.6_ increased by ∼60% due to Cu diffusion during sintering. The Cu/SS 304/Mg_2_Si_0.4_Sn_0.6_ contact fabricated by one-step hot press sintering had a specific contact resistance of ∼6.1 × 10^–6^ Ω·cm^2^ and remained <1 × 10^–5^ Ω·cm^2^ even after 15 days annealing at 723 K. Al and Ti interlayers were also used between the sputtering deposited Cu film and the Mg_2_Si semiconductor, but they weakened the electrode adhesion and increased the contact resistance.^148^

To reinforce the electrical conductivity of the Nb/Ta doped Mg_3_(Sb,Bi)_2_ TE leg, Fe, Mg turnings, and Cr powder were mixed and ball milled as TE interface powders materials.^44^ They were then sandwiched with Cu powders in a graphite die and sintered at 873 K for 10 min under a pressure of 50 MPa to produce a Cu/FeMgCr interfacial contact of 1.5 mm thick. The reduced interfacial barriers are conductive to carrier transport at low and high temperatures.

#### 4.3.2 Nickel Contact

Nickel has a relatively low contact resistance in the order of 10^–5^ Ω·cm^2^ and a closed CTE.^153^ It is commonly used as the electrode material for Mg_2_Si as it is durable and does not react significantly with Mg_2_Si at the working temperature.^71^ Direct hot-press bonding methods were used for leg preparation using Mg, Si, and Ni powders to form Mg_2_Si and nickel contact electrodes. The lowest contact resistances of Mg_2_Si samples with Ni electrodes was about 1.0 × 10^–5^ Ω·cm^2^. An intermediate layer consisting of different ternary phases of Mg/Si/Ni and Si_12_Ni_31_ was formed, which had good adhesion to both the Ni electrode and the Mg_2_Si.^154^ However, complex new phases formed at the Ni/Mg_2_Si interface complicated the relationship between processing and the contact resistance of the product.^155^ Although the joints had a decent shear strength of about 20–26 MPa, the contact resistance was also high at 1.28–1.44 × 10^–3^ Ω·cm^2^.^155^ After long-term annealing at 723 K for 600 h, the shear strength was 23 MPa while the contact resistance increased further to 3.7 × 10^–3^ Ω·cm^2^. These values are higher than the reported specific contact resistivity between Ni and commercial Mg_2_Si (∼10^–4^ Ω·cm^2^).^148,156^

The method of joining the presintered TE pellets with electrodes has an advantage over the co-sintering of TE and contact powder material together to fabricate the contact, as it allows for a disentanglement of the sintering temperature and the joining temperature. Interface thickness and composition can be tuned to reduce the contact resistance. A specific contact resistance (2.5–5 × 10^–5^ Ω·cm^2^) was obtained for the Ni electrode joined with both N- and P-type Mg_2_Si_1–*x*_Sn_*x*_ at a temperature lower than 973 K.^141^ Another method to obtain a better contact is achieved by pressing a thin nickel foil with powders instead of direct sintering of nickel powders on top of Mg_2_Si. The contact utilizing a nickel foil with Bi-doped Mg_2_Si TE powders was fabricated via an Induction Assisted Rapid Monoblock Sintering Technique, and the contact resistance was reduced to about 1.4 × 10^–5^ Ω·cm^2^.^157^ By pressing a thin nickel foil onto the surface of the leg during sintering, the contact resistance for Ni/Mg_2_Si_0.98_Bi_0.02_ junctions was only 5 × 10^–6^ Ω·cm^2^.^158^ Nevertheless, care must be taken, especially for Ni electrodes, as cracks are easily formed due to the brittle Mg_2_Si_1–*x*_Sn_*x*_ not accommodating well with Ni, given the slightly larger difference in CTE (17.5 vs 13 × 10^–6^ K^–1^).^141^ A mixture of the TE material (like Mg_2_Si_0.3_Sn_0.7_) and the contact metal powders (Cu/Ni) can be used as an intermediate diffusion barrier between the metal foil contact and TE materials.^159^ The electrical contact resistances of the joints compacted using the monoblock sintering technique were 3 and 19 × 10^–5^ Ω·cm^2^ for N- and P-type legs, respectively, and they remained stable after annealing at 673 K for 7 days. Mg_2_SiNi_3_ was also used as a diffusion barrier material between Ni and Mg_2_Si-based TE material.^160^ However, the migration of the Mg atom between Mg_2_Si to Mg_2_SiNi_3_ led to performance deterioration. Tohei et al. utilized aluminum instead of a silver-alloy braze to bond Mg_2_Si to Ni and reported a shear strength of 19 MPa; however, its thermal stability and contact resistance were not clear.^161^ Chromium layer was also used as a diffusion-impervious layer slowing down Ni diffusion and improving the mechanical properties of the contact.^162^

Nickel layers were electroplated onto the Mg_3_(Sb_1–*x*_Bi_*x*_)_2_ TE legs to facilitate the soldering and acting as an interfacial layer. However, the conductivity reduced after long service time.^163^ Nickel contact hot-pressed on the as-prepared Mg_3+δ_Bi_1.5_Sb_0.5_ had a resistance of 1.30 × 10^–5^ Ω·cm^2^, which increased to 1.85 × 10^–5^ Ω·cm^2^ after aging for 2100 h at 573 K.^163,164^ Mixtures of Ni with other metals like Fe or Cr also showed promising results, especially NiFe, which exhibited excellent thermal stability and the lowest ohmic contact resistance even after aging (1.30 × 10^–5^Ω·cm^2^) due to the formation of metallic NiMgBi between NiFe and Mg_3+δ_Bi_1.5_Sb_0.5_.

#### 4.3.3 Other Metals and Compounds

Silver nanoparticles soldered at low temperature (573 K) can sustain high service temperatures (>1000K), demonstrating stable performance and no degradation for various TE generators (such as Bi_2_Te_3_-based, PbTe-based, and half-Heusler-based) operating across a wide range of temperatures.^165^ Ag electrode joining at a temperature of 723 K with N- and P-type Mg_2_Si_1–*x*_Sn_*x*_ had a low specific contact resistance of 0.9–1.5 × 10^–5^ Ω·cm^2^.^141^ However, joining silver at higher temperatures like 873 K led to degraded samples due to the formation of a liquid phase. The formation of Ag defects in the solid solution lattice during the diffusion process also causes some concerns.^166^ Ag and α-MgAgSb have almost identical CTE (Table 4), which can thus reduce the interface stress significantly.^167^ Ag has excellent electrical and thermal conductivity and is easy to solder due to its softness; the small concentration gradient could decrease the elemental diffusion. A single α-MgAgSb leg with Ag pads on both the bottom and top surfaces was produced using a one-step hot-press technique, resulting in high mechanical strength and low resistance below 10^–5^ Ω·cm^2^.^168^

As an electrode or barrier material, silver is expensive; however, it has a low wetting contact angle, which helps the surface stick to the bonding alloys. A N-type segmented leg of Bi-doped Mg_2_Si (hot-side) bonded with Bi_2_Te_3_ (cold side) was fabricated via evaporation deposited 1 nm Ag and 50 nm Ti.^169^ In this design, silver (Ag) served as a “glue”, while titanium (Ti) enhanced adhesion between the semiconductors. The inclusion of Ti and Ag adhesive layers ensured lower contact resistance and strong bonding.

Aluminum is a poor dopant, with a larger CTE than the TE material. However, it is malleable, which helps to accommodate the mechanical stresses due to the CTE difference. Camut found that aluminum bonded well to P-type and N-type Mg_2_(Si,Sn), giving low electrical contact resistances (10 × 10^–6^ Ω·cm^2^), which were preserved or even lowered after annealing. The interface was clean and free of detrimental secondary phases.^151^ The low melting point of 933 K allows for direct bonding to the TE material. Al was used as a solder to join Mg_2_Si-based TE legs to Cu^148^ or Ni^161^ electrodes. The contact was established and mechanically strong, and no secondary phase was formed at the interface.

Iron demonstrated a lower contact resistance for Mg_3_Bi_1.5_Sb_0.5_ in comparison with Ni.^163^ It had a contact resistance of 1.18 × 10^–5^ Ω·cm^2^ after hot pressing, and increased to 1.76 × 10^–5^ Ω·cm^2^ after 2100 h aging at 573 K. Wu et al. employed alloys like Fe_7_Mg_2_Cr and Fe_7_Mg_2_Ti as interfacial contact material for Mg_3_Sb_2_-based TE legs, which had a balanced high σ_s_ (45–50 MPa) and low contact resistance of 2–3 × 10^–6^ Ω·cm^2^, although it increased to 7–8 × 10^–6^ Ω·cm^2^ after aging at 673 K for 15 days.^134^ An optimized interfacial alloy, FeCrTiMnMg, demonstrated a CTE of approximately 16 × 10^–6^ K^–1^ at room temperature, approaching that of Mg_3_Sb_1.5_Bi_0.5_. It had a contact resistance of 4 × 10^–6^ Ω·cm^2^, which remained stable after annealing at 673 K.

Skomedal et al. reported the bonding of a Mo electrode on the hot side of high-performance Mg_2_(Si_0.4_Sn_0.6_)_0.99_Sb_0.01_ and Mg_2_Si_0.53_Sn_0.4_Ge_0.05_Bi_0.01_, with thin layers of Pb, Ni, and Cr adopted to improve adhesion and contact.^153^ The best design exhibited an inner resistance in the region of 0.1 Ω at temperatures above 673 K.

Silicides such as TiSi_2_, CrSi_2_, CoSi, and NiSi exhibit low contact resistances with HMS in the order of 10^–6^–10^–5^ Ω·cm^2^. They align with the empirical “like-bonds-like” rule with Mg_2_Si-based TE. They were simultaneously sintered together with Mg_2_Si to form an electrode with Ni as a binder layer, and their contact resistance decreased by about a third compared to the standard Ni electrode.^170^ A maximum output power of 153 mW was reached with CoSi_2_ as the electrode, representing a 27% increase relative to the use of a nickel electrode at 600 K.

The CTE (22.1 × 10^–6^ K^–1^) of the Cu-based ternary alloy, Cu_2_MgFe, was closer to that of Mg_2_Sn_0.75_Ge_0.25_ (20.6 × 10^–6^ K^–1^) compared to Cu, Mg (29.5 × 10^–6^ K^–1^), and Fe (16.0 × 10^–6^ K^–1^), resulting in a high bonding strength of 15.1 MPa and a low contact resistance of 1.6 × 10^–5^ Ω·cm^2^ for a special designed Cu_2_MgFe/Mg_2_Sn_0.75_Ge_0.25_ interface.^142^ The stable interface was strengthened by reducing the chemical potential gradient and improving the diffusion activation energy barrier.

Other materials like Zirconium foil (16–125 μm) was used to bond skutterudite materials, acting as a diffusion barrier for Sb diffusion.^171^ However, whether this material can be used as a diffusion barrier on Sb containing Mg-based TE materials is still open for study.

#### 4.3.4 Fabrication and Selection of Metal Contact

There are various approaches to produce a contact layer on TE legs. For a thin layer (<10 μm), sputtering, electroplating, or chemical vapor deposition methods are employed to produce the contact material on a properly prepared TE leg. Each method offers different benefits in terms of precision, scalability, and cost. For a thick layer, hot pressing is applied to join the contact layer/foil to the TE material, which is relatively low cost and works effectively to create a diffusion barrier at elevated temperatures. If the two previously mentioned methods are not suitable, then conducting paste can be utilized to temporarily bond TE legs to the conducting strip.

In the testing of various metallic or silicide contacts with different Mg-based TE materials, Cu and Ni are commonly utilized as electrode or metalized materials. It has been observed that sintering Cu or Ni powder/plate separately with the TE pellets results in lower contact resistance. Additionally, interface engineering techniques such as transition or diffusion barrier layers may be employed to reduce contact resistance, prevent diffusion, and enhance the strength and durability of the electrode. Despite these advancements, the combined resistance from wiring and contact still contributes significantly, accounting for around 20–40% of the total module resistance. This poses a significant challenge and compromises the overall performance of the TE module. Consequently, achieving specific contact resistance below 1 × 10^–6^ Ω·cm^2^ remains a substantial challenge in optimizing contact layers for these materials.^71^

## 5 Progress of TEG Modules

**Figure 9:**
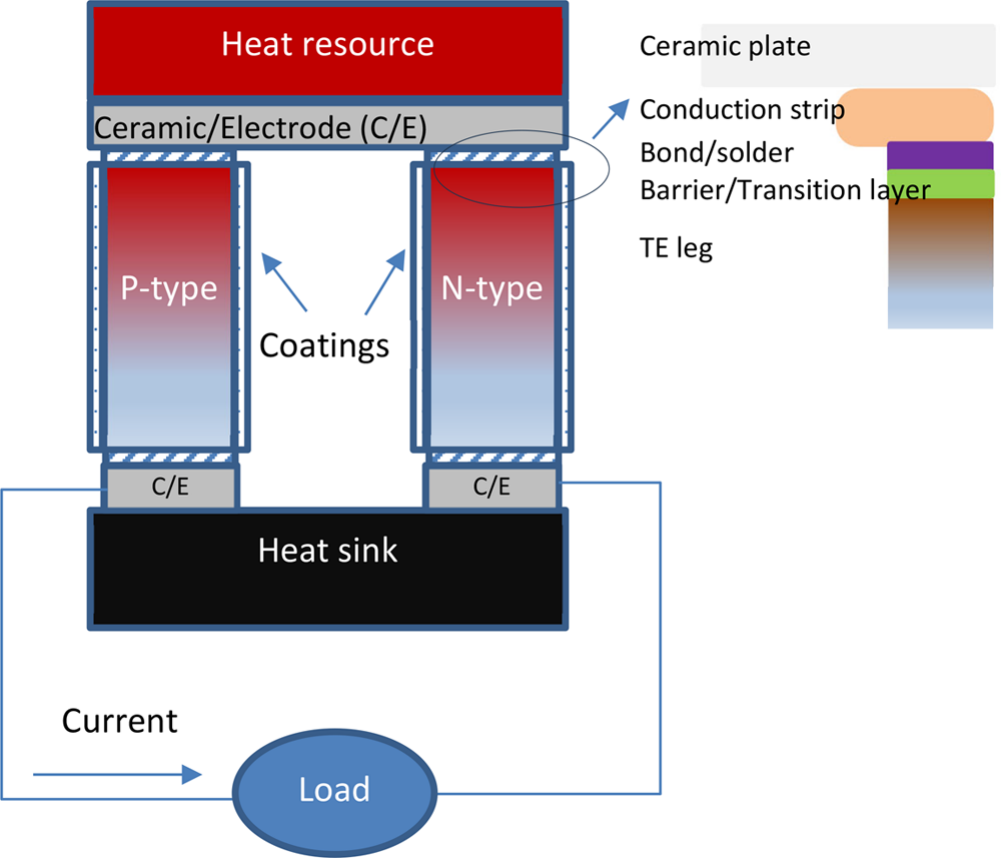
Configuration of a practical TE module and the joining stacking layers between the TE leg and the ceramic plate.

Thanks to their low cost, abundant resources, and ever-increasing performance, Mg-based TE devices are attracting growing interest and undergoing systematic investigations to overcome challenges for sustainable development toward higher power efficiency. In addition to optimizing the TE legs materials for the optimal performance, it is crucial to minimize degradation of TE device, especially over long periods, particularly on the hot side where interdiffusion and material loss are more prevalent. As illustrated in Figure 9, module design must prioritize approaches to improve stability of TE legs by either providing an inert environment or coating them. It is also important to reduce contact resistance and enhance joints by introducing functional layers between the TE leg and the current-collecting plate through metallization. These layers may include a diffusion barrier layer, a contact layer, an adhesion layer, and a compliant layer.

Currently, most of the research is concentrated on optimizing the TE properties; there is limited reporting on the TEG. TEGs require both N- and P-type materials to work efficiently, ideally with similar thermochemical and thermomechanical properties. In comparison with the well-developed N-type Mg_2_X(Si/Ge/Sn) materials with max ZT between 1.0 and 1.5, P-type Mg_2_X has much inferior performance with a max ZT just about 0.6 (Table 1), although it has a theoretical higher calculated max ZT value of 0.8.^18^ This mismatch requires TE modules to use Mg-based TE materials on a single leg or couple them with different TE materials such as high manganese silicide (HMS, max ZT of 1.0^176^) or MgAgSb^177^ (Table 1) to build TE generators.

Camut et al. produced a Mg_2_(Si,Sn)-based TEG with a power density of 0.9 W·cm^–2^ and 4% conversion efficiency at about 648 K.^174^ A single-leg Mg_2_Sn_0.75_Ge_0.25_ device using Cu_2_MgFe as contact layer exhibited a high power density of 2.6 W·cm^–2^ and conversion efficiency of 8% under a temperature difference of 643 K, which set a record-breaking value compared to other Mg_2_(Si, Ge, Sn)-based TE devices.^142^ A 8 mm thick N-type Nb/Ta doped Mg_3_(Sb,Bi)_2_ single leg bond with Cu contact by FeMgCr interface layer demonstrated a room-temperature power factor >30 μW·cm^–1^K^–2^. Thanks to the reduced lattice thermal conductivity, a record-high average ZT > 1.5 was achieved, resulting in a high TE conversion efficiency of 15%.^44^ An α-MgAgSb single leg fabricated by a one-step hot-press technique, with Ag pads on both the bottom and top surfaces, exhibited the highest TE conversion efficiency of 8.5% reported so far, operating between 293 and 518 K.^168^ At a current of 1.48 A, the power output reached 46.2 mW.^23^

A synergistic effort was undertaken to enhance the performance of Mg_3_Sb_1.5_Bi_0.5_ TE module by utilizing FeCrTiMnMg alloys as interface contact layer and a MgMn-based alloy as the protective coating.^134^ The output power density was 1.7 W·cm^–2^ and a conversion efficiency of 13% for the single-leg device was achieved at a temperature difference of 495 K between 278 K and 773 K. In combination with the p-type commercial Bi_2_Te_3_, a two-couple TE device can generate power of 0.8 W·cm^–2^ with an energy conversion efficiency of 6%.

A π-shaped TE module, comprising Mg_2_Si and high manganese silicide (HMS), were brazed to a Cu electrode using an Ag–Cu–Sn–Zn alloy, while the remaining segments of TE materials were soldered together using a Pb–Ag–Sn–In alloy. A high-power output of 42.9 W/kg was achieved under a 771 K temperature difference.^169^ Skomedal et al. developed a TEG device composed of N-type Mg_2_Si_0.4_Sn_0.6_ alloys and P-type material MnSi_1.75_Ge_0.01_. They utilized Pb, Ni, and Cr layers to increase the bond strength and reduce the contact resistance of Mo electrode on the hot side.^153^ The optimal module exhibited an average power output of 0.37 W and a maximum power output of 3.24 W at 1008 K, with an estimated efficiency as high as 5.3%. Additionally, with N-type Mg_2_Si_0.4_Sn_0.6_ and P-type MnSi_1.73_ both doped with Sb, the constructed TE module demonstrated an efficiency of more than 6.5% under a temperature difference of 520 K.^23^ In combination with N-type Mg_3_(Sb,Bi)_2_ and P-type α-MgAgSb TE legs, the conversion efficiency above 7% is achievable at medium temperature range such as 548 K^178^ and a record-high conversion efficiency of ∼7.3% is obtained at 593 K.^167^ The conversion efficiency of the latest developed Mg-based TE single legs or paired P/N modules is summarized in Table 5, compared with those commercial Bi_2_Te_3_, PbTe devices.

**Table 5:** Reported Conversion Efficiency of Mg-Based TE Single Leg or Paired Module (Comprising P/N Materials) Compared with Other Modules

	conversion efficiency (%)	max temperature (K)	temperature difference (K)	ref
Bi_2_Te_3_-based	6.6	575	235	(172)
PbTe	8.8	873	570	(173)
PbTe/Bi_2_Te_3_	11	873	590	(173)
Mg_2_Si_0.4_Sn_0.6_	4	648	375	(174)
Mg_2_Sn_0.75_Ge_0.25_	8	643	370	(142)
Mg_3.1_Sb_1.5_Bi_0.49_Te_0.01_	12.9	773	480	(175)
Mg_3_Sb_1.5_Bi_0.5_	13	773	495	(134)
Mg_3_Sb_1.5_Bi_0.49_Te_0.01_	15	790	497	(44)
a-MgAgSb	8.5	518		(168)
Mg_2_Si_0.4_Sn_0.6_/MnSi_1.73_	6.5	818	520	(23)
Mg_3_Sb_1.5_Bi_0.5_/α-MgAgSb	7.3	593		(167)
Mg_3_Sb_1.5_Bi_0.5_/Bi_0.4_Sb_1.6_Te_3_	6	448	150	(164)
Mg_3_Sb_1.5_Bi_0.5_/Bi_2_Te_3_ based	6	773		(134)
Mg_2_Sn_0.75_Ge_0.25_ /Bi_2_Te_3_	5.4	643	270	(142)

## 6 Future Perspectives

Although there has been significant progress in Mg-based TE materials and modules, ongoing efforts are still needed to further improve their performance and reliability. Some major areas for developments include:

1. Developing P- and N-type Mg-based compounds with matched ZT values by employing suitable alloying strategies and breakthrough fabrication methods/conditions to effectively manage the Fermi level and devise the band and phonon structures.
2. While various techniques are applied to enhance toughness without reducing transport properties, such as grain refinement and the addition of a second phase, the lack of experimental data on long-term durability, creep, and fatigue for practical application remains a challenge. Collecting a large amount of data for statistical analysis is necessary.
3. The long-term chemical stability of Mg-based TE materials and the power output stability of TE devices still face severe obstacles to conquer. Systematic studies are needed to ensure the future applicability of Mg-based TE materials.
4. The strength of joints between TE materials and electrodes affects the mechanical properties of TEGs. Optimization is needed to avoid thermal stress concentration and reduce the thickness of the diffusion layer. The geometry of TE legs and the method of arrangement in the module should be designed to restrict the movement of TE legs, for example, by using special frames or damping devices.
5. Advance in TE device fabrication is slower compared to the burgeoning fundamental research on TE materials, mainly due to the technical challenges associated with device design and assembly, particularly concerning electrode contact and interfacial design. Future efforts should involve holistic studies encompassing module design, material development, joint connection, fabrication method optimization, and sealing/protection of the Mg-based TE devices.
6. A life cycle strategy from module design, materials choice, TE leg production, TEG assembly, and recycling should be proposed to conserve materials and resources for the benefit of the environment.

## Data Availability

The data that support the findings of this study are available from the corresponding author upon reasonable request.

## Abbreviations Used:

ALD: Atomic Layer Deposition
CTE: Coefficient of Thermal Expansion
CVD: Chemical Vapor Deposition
DC: Direct Current
EDS: Energy-Dispersive X-ray Spectrometry
HMS: High Manganese Silicide
MS: Magnetron Sputtering
PVD: Physical Vapor Deposition
RF: Radio Frequency
SDC: Samaria-doped Ceria
SEM: Scanning Electron Microscopy
SPS: Spark Plasma Sintering
TE: Thermoelectric
TEG: Thermoelectric Generator
TGA: Thermogravimetric Analysis
XRD: X-ray Diffraction
YSZ: Yttria Stabilized Zirconia
ZT: Figure of Merit
